# Ambulatory medical services utilization for menstrual disorders among female personnel of different medical professions in Taiwan: a nationwide retrospective cohort study

**DOI:** 10.1186/s12905-015-0220-3

**Published:** 2015-08-26

**Authors:** Malcolm Koo, Chien-Han Chen, Kun-Wei Tsai, Ming-Chi Lu, Shih-Chun Lin

**Affiliations:** Department of Medical Research, Dalin Tzu Chi Hospital, Buddhist Tzu Chi Medical Foundation, Dalin, Chiayi Taiwan; Dalla Lana School of Public Health, University of Toronto, Ontario, Canada; Division of Obstetrics and Gynecology, Dalin Tzu Chi Hospital, Buddhist Tzu Chi Medical Foundation, Chiayi, Taiwan; Division of Geriatrics, Dalin Tzu Chi Hospital, Buddhist Tzu Chi Medical Foundation, 2 Minsheng Road, Dalin, Chiayi 62247 Taiwan; Division of Allergy, Immunology and Rheumatology, Dalin Tzu Chi Hospital, Buddhist Tzu Chi Medical Foundation, Dalin, Chiayi Taiwan; School of Medicine, Tzu Chi University, Hualien, Taiwan

## Abstract

**Background:**

Menstrual disorders and their adverse symptoms can have a deleterious effect on both the private and working lives of women. Previous studies indicated that female nurses have elevated risk of menstrual disorders. Moreover, female nurses showed a higher incidence of ambulatory care visit for genitourinary diseases compared with other female medical personnel. However, little is known whether the medical services utilization for menstrual disorders were different among personnel from various medical professions. Therefore, the present study compared the ambulatory medical services utilization for menstrual disorders among personnel of six different medical professions in Taiwan using a nationwide, population-based health claim research database.

**Methods:**

The National Health Insurance Research Database (NHIRD) was used to identify female medical professionals, aged 18 to 45 years, who obtained their licenses during January 1, 2000 to December 31, 2012. Personnel from six different medical professions were examined and they included (1) medical technologists and therapists, (2) registered nurses, (3) physicians, (4) doctors of Chinese medicine, (5) dentists, and (6) pharmacists. Diagnoses of menstrual disorders, based on International Classification of Diseases, Ninth Revision, Clinical Modification (ICD-9-CM) codes, were obtained from the ambulatory medical services utilization that occurred after their license date. Cox proportional hazards model was used to assess the hazards of medical services utilization for menstrual disorders using medical technologists and therapists as the reference category.

**Results:**

A total of 7653 medical personnel were included in the analysis. Using the group containing medical technologists and therapists as the reference category, registered nurses (adjusted hazards ratio [AHR] = 1.13, *p* = 0.018) and doctors of Chinese medicine (AHR = 2.52, *p* < 0.001) showed a significant increased risk of medical services utilization for menstrual disorders. Conversely, physicians showed a significant decreased risk of medical services utilization for menstrual disorders (AHR = 0.58, *p* < 0.001). Regarding the nine specific menstrual disorders observed in this study, registered nurses and doctors of Chinese medicine showed an increased risk in six and four of them, respectively. Pharmacists showed an increased risk only in menorrhagia (AHR = 1.64, *p* = 0.020) and dentists showed no significant differences in any of the nine specific menstrual disorders compared with medical technologists and therapists. Physicians showed a significant decreased risk all specific menstrual disorders except menorrhagia and dysfunctional uterine bleeding.

**Conclusions:**

Findings from this population-based cohort study revealed that, compared with medical technologists and therapists, registered nurses and doctors of Chinese medicine exhibited significant increased risks in medical services utilization for menstrual disorders whereas physicians showed a significant decreased risk in menstrual disorders. Further studies should be conducted to delineate whether the differences in the medical services utilization is an indicator of risk of menstrual disorders or the results of varying patterns of health care seeking behavior among women of different medical professions.

## Background

Regular menstruation is a sign of normal ovarian function and indicative of a positive reproduction health of a woman. However, menstrual problems such as dysmenorrhea, irregular menstrual cycles, and premenstrual symptoms are common complaints among women of reproductive age. These problems can impact on the daily routines including work performance and social activities. A large-scale online survey on 19,254 women in Japan found that 74 % of the respondents had suffered from menstrual symptoms. The estimated annual economic burden was 8.6 billion US$ for the Japanese female population [1]. A cross-sectional study based on self-administered questionnaire on 598 nurses in Taiwan revealed that the lifetime prevalence of premenstrual discomfort was 84.5 %. Although the response rate was only 44 %, the authors claimed that the socio-economic characteristics of the sample were comparable with the source population [2]. In addition, a nationwide study based on the United States National Health Interview Survey 1999 found that women who have a heavier menstrual flow were 1.5 times as likely to use health care as women who had a lighter or normal flow [3]. Work loss as a consequence of increased menstrual flow was estimated to be US$1692 annually per woman [4]. Furthermore, menstrual cycle irregularity before middle age had been shown to increase the risk of cardiovascular disease [5], type 2 diabetes [6], and osteoporosis [7]. Amenorrhea that is not the normal results of pregnancy or menopause, not only can affect fertility, but it can also impact the self-esteem of a woman [8].

Work stress and shift work are possible factors that can adversely affect the menstrual function of women and therefore, represent an occupational health challenge for female nurses [9]. Working in emergency room and working rotating shift were also found to be significantly associated with irregular ovarian cycle pattern in a study of 151 female nurses [10]. In a study of 493 female nurses in Thailand, the prevalence of dysmenorrhea was found to be over 70 %. Daily activities and quality of life were negatively impacted among nurses who had moderate to severe dysmenorrhea [11]. In addition, a cross-sectional study of 435 female nurses from five regional hospitals in Taiwan revealed that 57 % of the nurses had poor sleep quality. Scores for poor sleep quality were associated with premenstrual dysphoria, occupational injury, illness, and medication use [12]. Labyak and colleagues [13] suggested that changes in menstrual function might be a marker of shift work intolerance. Irregular menstrual patterns were also found to be associated with rotating shift work, which might affect fertility and other cycle-related aspects of the nurses' health [14].

Menstrual disorders and their adverse symptoms can have a deleterious effect on both the private and working lives of females. Nevertheless, limited population-based studies are available that examine the risk of menstrual disorders among female personnel of different medical professions. A retrospective cohort study using the data from the Taiwan National Health Insurance Research Database (NHIRD) reported that female nurses had significantly elevated incidence of genitourinary diseases (adjusted rate ratio = 1.20) compared with other female medical personnel [15]. Based on the same claim based dataset, the present nationwide study further investigated the risks of medical services utilization for various menstrual disorders among female personnel of six different medical professions.

## Methods

### Study population

The Taiwan NHIRD contains comprehensive administrative and claim data from the National Health Insurance program, which is a mandatory single-payer social health insurance system implemented in Taiwan beginning in 1995 [16]. We used the registry for medical personnel of the NHIRD to identify female licensed medical personnel who were in practices during 2000 to 2012. Personnel from six different medical professions were included in this study and they included (1) medical technologists and medical technologist assistants, physiotherapists and physiotherapist assistants, occupational therapists and occupational therapist assistants, medical radiological technologists and medical radiological technicians (hereinafter referred to as medical technologists and therapists), (2) registered nurses, (3) physicians, (4) doctors of Chinese medicine, (5) dentists, and (6) pharmacists and assistant pharmacists. Subjects aged less than 18 years or above 45 years were excluded from the analyses. We then used the Longitudinal Health Insurance Database 2000 (LHID2000) of the NHIRD to obtain their utilization of outpatient care services that occurred between their licensing date and December 31, 2012. The LHID2000 contains medical claims and registration files for one million enrollees, who were randomly selected from all 23.7 million enrollees listed in the 2000 Registry of Beneficiaries under the National Health Insurance program.

### Outcome measures

Diagnosis of menstrual disorders were identified based on the International Classification of Diseases, Ninth Revision, Clinical Modification (ICD-9-CM) and they included amenorrhea (ICD-9-CM code 626.0), oligoemorrhea or hypomenorrhea (ICD-9-CM code 626.1), menorrhagia (ICD-9-CM code 626.2), irregular menstrual cycle (ICD-9-CM code 626.4), metrorrhagia (ICD-9-CM code 626.6), dysfunctional uterine bleeding (ICD-9-CM code 626.8), unspecified disorders of menstruation and other abnormal bleeding from female genital tract (ICD-9-CM code 626.9), dysmenorrhea (ICD-9-CM code 625.3), and premenstrual tension syndrome (ICD-9-CM code 625.4).

### Statistical analysis

Cox proportional hazards regression analyses was used to compare the hazards of medical services utilization for menstrual disorders in six medical professions using the group medical technologists and therapists as the reference category. The group of medical technologists and therapists was chosen as the reference group because while they have presumably similar convenient access to medical services as the other groups, they generally do not have to work in rotating shifts. Ten models were fitted (one for any menstrual disorders and nine for each of the specific menstrual disorder). All models were adjusted for licensing age. Person-years of observation were calculated starting when the medical personnel became licensed and followed to the date of a menstrual disorder diagnosis. For subjects without any diagnosis of menstrual disorders, their follow-up time was censored on the date of their last ambulatory medical utilization in the data set. The non-proportionality assumption was evaluated by including a product term between medical profession and time in the model. A two-sided *p* value < 0.05 was considered statistically significant. All analyses were performed using IBM SPSS Statistics software package, version 21.0 (IBM Corp., Armonk, NY, USA).

Since the NHIRD files contain only de-identified secondary data, the need for informed consent from individual subjects was waived. The study protocol was reviewed and approved by the institutional review board of the Dalin Tzu Chi Hospital, Buddhist Tzu Chi Medical Foundation, Taiwan (No. B10202020).

## Results

A total of 7653 female personnel were included in the analysis. The mean (standard deviation) licensing age was 27.0 (6.0) years. The median length of follow-up for any menstrual disorders was 653 days and for the specific menstrual disorders ranged from 1383 days in irregular menstrual cycle to 2281 days in premenstrual tension syndrome.

Using the group containing medical technologists and therapists as the reference category, registered nurses (adjusted hazards ratio [AHR] = 1.13, *p* = 0.018) and doctors of Chinese medicine (AHR = 2.52, *p* < 0.001) showed a significant increased risk of medical services utilization for menstrual disorders (Table 1). Conversely, physicians showed a significant decreased risk of medical services utilization for menstrual disorders (AHR = 0.58, *p* < 0.001).

**Table 1:** Results of the Cox proportional hazards regression analyses of medical services utilization for menstrual disorders among different medical professions in Taiwan (*N* = 7653)

Condition	Adjusted hazards ratio (95 % confidence interval)					
(ICD-9-CM code)	*p value*					
	MT, OT, PT, RT	Registered nurses	Physicians	Doctors of Chinese medicine	Dentists	Pharmacists
	(*n* = 658)	(*n* = 5949)	(*n* = 238)	(*n* = 48)	(*n* = 101)	(*n* = 479)
Any menstrual disorder	1.00	1.13 (1.02-1.24)	0.58 (0.48-0.72)	2.52 (1.81-3.52)	1.00 (0.77-1.30)	0.96 (0.83-1.12)
		*0.018*	*<0.001*	*<0.001*	*0.988*	*0.589*
Amenorrhea (626.0)	1.00	1.26 (1.08-1.47)	0.58 (0.41-0.84)	0.84 (0.41-1.70)	1.00 (0.65-1.55)	*0.85 (0.66-1.09)*
		*0.004*	*0.003*	*0.620*	*>0.999*	*0.192*
Oligoemorrhea or hypomenorrhea (626.1)	1.00	1.34 (0.98-1.82)	0.28 (0.10-0.76)	4.71 (2.35-9.41)	0.33 (0.08-1.38)	1.34 (0.86-2.09)
		*0.066*	*0.013*	*<0.001*	*0.129*	*0.191*
Menorrhagia (626.2)	1.00	1.44 (1.05-1.98)	0.95 (0.52-1.71)	1.02 (0.32-3.28)	0.97 (0.41-2.28)	1.64 (1.08-2.49)
		*0.023*	*0.855*	*0.980*	*0.937*	*0.020*
Irregular menstrual cycle (626.4)	1.00	1.27 (1.11-1.46)	0.49 (0.36-0.68)	1.32 (0.80-2.16)	0.87 (0.59-1.28)	1.08 (0.89-1.33)
		*<0.001*	*<0.001*	*0.276*	*0.473*	*0.438*
Metrorrhagia (626.6)	1.00	1.32 (1.00-1.73)	0.25 (0.10-0.63)	not calculable	1.20 (0.60-2.43)	0.98 (0.64-1.50)
		*0.048*	*0.003*		*0.608*	*0.920*
Dysfunctional uterine bleeding (626.8)	1.00	1.34 (1.10-1.64)	0.72 (0.47-1.11)	1.00 (0.44-2.28)	1.52 (0.95-2.43)	0.89 (0.65-1.22)
		*0.004*	*0.134*	*0.996*	*0.080*	*0.463*
Unspecified disorders of menstruation (626.9)	1.00	1.22 (1.04-1.44)	0.71 (0.50-1.01)	2.69 (1.67-4.34)	0.91 (0.57-1.45)	1.14 (0.89-1.45)
		*0.016*	*0.054*	*<0.001*	*0.695*	*0.302*
Dysmenorrhea (625.3)	1.00	0.96 (0.81-1.14)	0.57 (0.38-0.85)	5.18 (3.40-7.90)	0.68 (0.39-1.18)	0.86 (0.66-1.13)
		*0.625*	*0.006*	*<0.001*	*0.169*	*0.288*
Premenstrual tension syndrome (625.4)	1.00	0.74 (0.52-1.06)	0.23 (0.07-0.76)	3.74 (1.72-8.13)	0.19 (0.03-1.41)	0.69 (0.38-1.25)
		*0.099*	*0.015*	*0.001*	*0.105*	*0.219*

All Cox regression models were adjusted for licensing age

*ICD-9-CM* International Classification of Diseases, Ninth Revision, Clinical Modification; *95 % CI* 95 % confidence interval; *MT* medical technologists and medical technologist assistant; *OT* occupational therapists and occupational therapist assistants; *PT* physiotherapists and physiotherapist assistants; *RT* medical radiological technologists and medical radiological technicians

Regarding the nine specific menstrual disorders observed in this study, registered nurses showed an increased risk in six of them, including amenorrhea, menorrhagia, irregular menstrual cycle, metrorrhagia, dysfunctional uterine bleeding, and unspecified disorders of menstruation. Doctors of Chinese medicine showed an increased risk in four of the specific menstrual disorders, including oligoemorrhea or hypomenorrhea, unspecified disorders of menstruation, dysmenorrhea, and premenstrual tension syndrome. Pharmacists showed an increased risk only in menorrhagia and dentists showed no significant differences in any of the nine specific menstrual disorders compared with medical technologists and therapists. Physicians showed a significant decreased risk in medical services utilization in all nine specific menstrual disorders except menorrhagia and dysfunctional uterine bleeding.

## Discussion

This retrospective, population-based, cohort study found that the risks of medical services utilization of a number of specific menstrual disorders were significantly increased in registered nurses and doctors of Chinese medicine compared with the reference group of medical technologists and therapists. Conversely, the risk of medical services utilization for menstrual disorders in physicians was significantly decreased. In addition, the risks of any menstrual disorders in dentists and pharmacists were similar to the reference group. To our knowledge, this is the first population-based study that compared the medical services utilization for menstrual disorders among female personnel of different medical professions. Most previous studies focused mainly on nurses.

Previous studies have shown that nurses had a high prevalence of menstrual disorders including dysmenorrhea [11, 17, 18], irregular menstrual patterns [10], and premenstrual symptoms [19]. A cross-sectional study on 746 nurses in Taiwan reported self-perceived high job stress was significantly associated with irregular menstrual cycles and longer menstrual bleeding periods [20]. The present study revealed that the risks of medical services utilization for amenorrhea, menorrhagia, irregular menstrual cycle, metrorrhagia, dysfunctional uterine bleeding, and unspecified disorders of menstruation were also significant elevated among nurses compared with the reference group. However, the risks of medical services utilization for dysmenorrhea and premenstrual tension syndrome were not different from the reference group. The differences between our study and the previous studies might be attributed to the choice of different reference group. To minimize the possible bias introduced by the convenience in accessing to medical services among medical personnel, we did not use the general population as the reference group. Instead, we used the group consisted of four professions including (1) medical technologists and medical technologist assistants, (2) physiotherapists and physiotherapist assistants, (3) occupational therapists and occupational therapist assistants, and (4) medical radiological technologists and medical radiological technicians as the reference group because these individuals should have similar access to medical services compared with those in the other five medical professions.

Our study also observed that the risk of medical services utilization for menstrual disorders in doctors of Chinese medicine was significantly increased compared to that in the reference group. The magnitude of the adjusted hazards ratios ranged from 2.7 in unspecified disorders of menstruation to 5.2 in dysmenorrhea. Since there is no previous research indicating that doctors of Chinese medicine have increased risk of menstrual disorders, it is likely that the elevated hazards ratios is a reflection of their unique medical seeking behavior. Under the framework of Chinese medicine, excessive or scanty menstrual flow, dysmenorrhea, premenstrual symptoms, and irregular menstrual cycles are considered as diagnostic signs that represent underlying pattern of disharmony of the body. Various traditional Chinese medicine modalities based on acupuncture, moxibustion, and Chinese herbal formula have been traditionally available and are still commonly used for rebalancing any disharmonies [21]. A NHIRD study based on the traditional Chinese medicine outpatient claims from 1996 to 2001 showed that traditional Chinese medicine was widely used in Taiwan. Disorders of menstruation and abnormal bleeding ranked the 13th most common disease diagnosis among the 156 millions traditional Chinese medicine visits from 1996 to 2001 [22]. Since traditional Chinese medicine is well accepted by the general population for the treatment of menstrual disorders, doctors of Chinese medicine should especially rely on it.

In contrast to the doctors of Chinese medicine, physicians of Western medicine showed a significant decreased risk of medical services utilization for menstrual disorders. The exact reasons for the decreased hazard ratios are not clear. One possible reason is that the lower hazards are the result of a bias similar to the healthy hire effect, where women in poor general or reproductive health may be less likely to consider medicine as their career choice. Another more likely reason is the differences in the self-care behavior of medical personnel [18]. Since little can be done medically for many menstrual disorders except for the use of analgesics to reduce pain, female physicians may choose to take care of their own menstrual disorders rather than to seek formal medical care compared to medical technologists and therapists. A population-based cohort study followed 5,815,781 young Taiwanese women revealed that female physicians were the least likely to have undergone at least one Pap smear test during the three-year study period, compared with female relatives of physicians, and general women of similar socio-economic background [23]. Similarly, another study based on the NHIRD found that Pap-test utilization were highest for pharmacists (58.1 %) and nurses (58.0 %) but lowest for physicians (48.6 %) [24]. Whether the lower medical services utilization for menstrual disorders observed among physicians in this study can be attributed to cultural and occupational concerns similar to the situation regarding cervical cancer screening utilization will require further studies.

The main strengths of our study are its sample size and the population-based design. Nevertheless, several potential study limitations should be noted. First, menstrual disorders can have multiple etiologies but due to the inherent limitations of the data source, it was not possible to adjust for factors such as types of work shift, levels of job stress, and body mass index in the analyses. Second, all diagnoses were based on ICD-9-CM codes in the claim records and the possibility of misclassification cannot be completely ruled out.

## Conclusion

In conclusion, compared with medical technologists and therapists, registered nurses and doctors of Chinese medicine exhibited significant increased risks in medical services utilization for menstrual disorders whereas physicians showed a significant decreased risk. Menstrual disorders can reduce quality of life and lead to a loss of productivity at work. Our findings have important implications for management of medical personnel and further studies should be conducted to delineate whether the differences in the medical services utilization is an indicator of risk of menstrual disorders or the results of varying patterns of health care seeking behavior among women of different medical professions.

## Abbreviations

NHIRD: National Health Insurance Research Database
ICD-9-CM: International Classification of Diseases, Ninth Revision, Clinical Modification
95 % CI: 95 % Confidence Interval
HR: Hazards ratio
